# The knowledge, attitude and behavior of ICU nurses regarding ICU-acquired weakness: a cross-sectional survey

**DOI:** 10.1186/s12912-024-01942-9

**Published:** 2024-06-04

**Authors:** Mingfeng Zhao, Anyun Qiu, Zhijing Zhang, Fang Pan, Yongxia Gao

**Affiliations:** 1 Department of Emergency and Critical Care Medicine, The First Affiliated Hospital of Nanjing Medical University, Nanjing, 210029 China; 2 Department of general surgery, Children’s Hospital of Nanjing Medical University, Nanjing, 210000 China; 3 Department of otorhinolaryngology, Children’s Hospital of Nanjing Medical University, Nanjing, 210000 China

**Keywords:** Care, Clinical, ICU-acquired weakness, Intensive care unit, Nurse, Survey

## Abstract

**Background:**

Intensive care unit-acquired weakness (ICU-AW) is very common in ICU patients. It is important to understand the status quo of knowledge, attitude and behavior of ICU nurses about ICU-AW. This survey aimed to investigate the knowledge, attitude and behavior of ICU nurses about ICU-AW, to provide useful implications for clinical care.

**Methods:**

ICU nurses from two tertiary hospitals in China from October 10 to November 15, 2023 were included. The ICU-AW knowledge, attitude and behavior questionnaire of ICU nurses with 31 items were used for survey. SPSS24.0 statistical software was used for data analysis.

**Results:**

A total of 364 ICU nurses were included for survey. The ICU-AW knowledge of ICU nurses was 21.96 ± 5.72 (< 50% of the total knowledge score), the ICU-AW attitude of ICU nurses was 30.24 ± 5.05(< 75% of the total attitude score), the ICU-AW behavior of ICU nurses was 26.77 ± 5.81(< 75% of the total behavior score), the total score was 79.21 ± 12.69(< 75% of the total score). Nurses’ ICU-AW knowledge, attitude and behavior were all correlated (all *P* < 0.05). Multiple linear regression analyses indicated that age, years of ICU work experience, professional ranks and titles, had received the training about the ICU-AW were the influencing factors of knowledge, attitude and behavior of ICU nurses about ICU-AW (all *P* < 0.05).

**Conclusions:**

The knowledge, attitude and behavior of ICU nurses’ ICU-AW needs to be improved, and there are many influencing factors. Hospital nursing administrators should strengthen the training of nurses’ ICU-AW knowledge and improve the cognitive and practical ability of ICU nurses on ICU-AW, so as to reduce the occurrence of ICU-AW.

**Supplementary Information:**

The online version contains supplementary material available at 10.1186/s12912-024-01942-9.

## Introduction

Intensive care unit-acquired weakness (ICU-AW) refers to that critically ill patients are treated in ICU for a long time, especially in mechanical ventilation, resulting in a significant decrease in muscle mass and muscle strength [1]. ICU-AW is characterized by symmetrical involvement of the extremities especially the lower limbs, the most severe muscle weakness in the neuromuscular areas of the proximal extremities (such as shoulder and waist), and the respiratory muscles may also be involved, resulting in delayed weaning of mechanical ventilation [2–4]. The incidence of ICU-AW is 67%, which is still as high as 36% after discharge [5]. Previous study [6] has reported that early signs of ICU-AW will appear from the second day of admission to ICU. ICU-AW will not only affect the rehabilitation of patients, prolong the length of ICU stay of patients, increase the cost of medical treatment, but also reduce the quality of life and survival rate of patients [7–9]. ICU health care providers should not only maintain the cardiopulmonary, digestive and renal functions of critically ill patients to ensure the survival rate of patients, but also master ICU-AW-related knowledge to minimize the occurrence of ICU-AW and improve the ability and quality of life of critically ill patients [10, 11].

Some studies [12, 13] suggest that the poor development of early activities in ICU may be due to the lack of attention of clinical nurses, doctors, hospital administrators and so on. The results of previous study [14] show that medical staff are lack of understanding of the causes of ICU-AW and have not clear measures to prevent the occurrence of ICU-AW. There are also researchers who have intervened critically ill patients with early activities and achieved good results. Ye et al [15] have used a self-designed bed rest exercise therapy device to carry out active functional exercise on the second day after admission of ICU patients, which have promoted the rehabilitation of critically ill patients and have reduced the incidence of ICU-AW. At present, little attention has been paid to ICU-AW, and effective prevention and treatment measures are lacking. Therefore, in the daily medical and care services of ICU, it is very necessary to deepen the understanding of ICU-AW and have the behavior of preventing ICU-AW. Therefore, this study aims to investigate the current situation of ICU nurses’ ICU-AW knowledge, attitude and behavior, and analyze the main influencing factors, to provide insights for clinical nursing care.

## Methods

### Study design

This study was a cross-sectional survey design.

### Ethical consideration

This study was a cross-sectional survey design. The study had been reviewed and approved by the ethics committee of The First Affiliated Hospital of Nanjing Medical University (approval number: 2024-SR-007) and Children’s Hospital of Nanjing Medical University (approval number: 202402013-1). Written informed consents had been obtained from all the surveyed nurses.

### Participants

ICU nurses from the comprehensive medical-surgical ICUs of two tertiary hospitals in Jiangsu province were included as participants from October 10 to November 15, 2023. The inclusion criteria of this survey were as follows: ICU registered nurses who directly provided nursing services for critically ill patients; working independently for more than 6 months in ICU; nurses agreed to participate in this study and signed informed consent form. The exclusion criteria for nurses were as follows: nurses who were on maternity leave, sick leave, rotation or other reasons who were not on duty; nurses who did not want to participate in this survey.

### Survey tool

The contents of the questionnaire included the general data of nurses and the questionnaire of ICU-AW knowledge, attitude and behavior of ICU nurses. The general information we intentionally to collect in this survey included ICU nurses’ gender, age, education level, marital status, years of ICU work experience, professional ranks and titles, and whether the ICU nurse had received the training about the ICU-AW. The ICU-AW knowledge, attitude and behavior questionnaire of the ICU nurses had been developed by Wu et al [16], which was divided into three dimensions: knowledge, attitude and behavior of ICU-AW, with 31 items. (1) ICU-AW knowledge (15 items): subjective knowledge (items 1–6), three options for answers, respectively, 3 points, 2 points and 1 point; objective knowledge (items 7–15), three options for answers, 3 points, 2 points and 1 point respectively. The total score range was 15 ∼ 45 points, the higher the score, the better the ICU-AW knowledge of ICU nurses. (2) ICU-AW attitude (8 items), there were five options for answers, which were 5, 4, 3, 2 and 1 points respectively. The total score ranges from 8 to 40, the higher the score, the better the ICU-AW attitude of ICU nurses. (3) ICU-AW behavior (8 items), the answer had five options, with a total score of 5, 4, 3, 2 and 1, respectively. The total score ranged from 8 to 40, and the higher the score, the more ICU-AW behaviors performed by ICU nurses. We made a quartile discrimination of the scores of each part. <25% indicated very poor, 25 ∼ 50% indicated poor, 50%∼75% indicated normal, > 75% indicated good. It has been reported that the Cronbach’s α coefficient of the ICU-AW knowledge, attitude and behavior questionnaire was 0.858 and the content validity was 0.873, indicating that the questionnaire had good reliability and validity [17, 18].

### Survey process

We had performed a pilot study on ten nurses before carrying on the survey process to assess the clarity, feasibility, and time needed to fill out the questionnaire. When the researchers handed out the questionnaire, they used unified instructions for guidance and requirements. The questionnaire was filled out by ICU nurses independently, and each questionnaire was completed within 40 min, and was collected on the spot. The researchers carefully checked the recovered ICU-AW knowledge, attitude and behavior questionnaire of ICU nurses. If the items were filled in an option, or if the missing items in the same questionnaire were more than 20%, the questionnaire would be considered invalid and would not be entered into the database.

In order to better count and checked the questionnaire data, the recovered questionnaires were uniformly numbered. In this study, the data and data were checked and input twice. If there was any logical error, we checked and corrected the original data to ensure the accuracy and authenticity of the data.

### Statistical analysis

According to previous research reports [19, 20], the total sample size should be 5 ∼ 10 times of the variable number, that is, sample size = variable × (5 ∼ 10) × [1 + (10%∼15%)]. There were 31 independent variables in this study, and the calculated sample size was 155 ∼ 310 people. Considering that there may be invalid questionnaires in the course of the survey, the sample size could be expanded to 310 × 110%=341. Therefore, at least 341 ICU nurses should be included in this survey.

SPSS24.0 statistical software was used for data processing and analysis. Frequency and constituent ratio were used to describe the general data of ICU nurses. All the data has been checked for normality by Shapiro-Wilk test before comparison, mean ± standard deviation or median and interquartile range were used to describe the scores of ICU-AW knowledge, attitude and behavior of ICU nurses. The comparison of each knowledge, attitude and behavior scores were performed by independent t-test or analysis of variance test as appropriated. Pearson correlation was used to analyze the correlation among ICU-AW knowledge, attitude and behavior of ICU nurses (| r| < 0.3 indicates no correlation, 0.3 < =| r| < = 0.5 indicates low correlation, 0.5 < =| r| < = 0.8 indicates significant correlation, and| r|> 0.8 indicates high correlation). Regularization terms were introduced to help correct multicollinearity, it encouraged smaller coefficient values, and effectively reduced the sensitivity of the model to relevant predictive variables [21]. This regularization could lead to more stable and explainable regression coefficients, and could improve the overall performance of the model in the presence of multicollinearity [22]. Multiple linear regression analysis was used to evaluate the influencing factors of ICU-AW knowledge, attitude and behavior scores. All the tests in this study were bilateral tests, *P* < 0.05 suggested that there was significant statistical difference.

## Results

### Clinical characteristics of included nurses

A total of 380 questionnaires were distributed initially, of which 16 were invalid and a total of 364 valid questionnaires were included. As presented in Table 1, most of the ICU nurses investigated were female, with an average age of 30.15 ± 6.22 years old, and an average of 5.25 ± 2.08 ICU working years. Most ICU nurses were senior nurses with junior college or bachelor degree, and most nurses were married. Only 36.54% nurses had received the training about the ICU-acquired weakness.

**Table 1:** The characteristics of included nurses(*n* = 364)

Items	Number of nurses	Percentage
Gender		
Male	56	15.38%
Female	308	81.62%
Age(y)		
18 ∼ 25	131	35.99%
26 ∼ 35	144	39.56%
36 ∼ 45	81	22.25%
> 45	8	2.20%
Education level		
Junior college degree	187	51.37%
Bachelor degree	171	46.98%
Master degree	6	1.65%
Marital status		
Unmarried	148	40.66%
Married	216	59.34%
Years of ICU work experience		
< 5	114	31.32%
6 ∼ 10	178	48.90%
11 ∼ 20	59	16.21%
> 20	13	3.57%
Professional ranks and titles		
Junior nurse	125	34.34%
Senior nurse	176	48.35%
Nurse in charge	55	15.11%
Deputy chief nurse	5	1.37%
Chief nurse	3	0.82%
Had received the training about the ICU-acquired weakness		
Yes	133	36.54%
No	231	63.46%

### Results of each KAB score

As indicated in Table 2, the ICU-AW knowledge of ICU nurses was 21.96 ± 5.72 (< 50% of the total knowledge score), the ICU-AW attitude of ICU nurses was 30.24 ± 5.05(< 75% of the total attitude score), the ICU-AW behavior of ICU nurses was 26.77 ± 5.81(< 75% of the total behavior score), the total score was 79.21 ± 12.69(< 75% of the total score).

**Table 2:** The knowledge, attitude and behavior scores of nurses about the about ICU-acquired weakness

Item	Average score	Score range
Knowledge	21.96 ± 5.72	14∼25
Attitude	30.24 ± 5.05	22∼36
Behavior	26.77 ± 5.81	20∼29
Total score	79.21 ± 12.69	66∼90

### Correlation analysis in nurses’ characteristics and knowledge, attitude and behavior

As presented in Table 3, nurses’ ICU-AW knowledge, attitude and behavior were all positively correlated (*r* = 0.548, 0.605 and 0.566, all *P* < 0.05).

**Table 3:** Correlation analysis of nurses’ knowledge, attitude and behavior about ICU-acquired weakness

Item	Knowledge score	Attitude score	Behavior score
Knowledge score	1.00	0.548^*^	0.605^*^
Attitude score	-	1.00	0.566^*^
Behavior score	-	-	1.00

*, *P* < 0.05

### Comparison of scores in characteristics in univariate analysis

As shown in Table 4, univariate analysis indicated that there were statistical differences in the knowledge score of ICU nurses with different gender, age, education level, years of ICU work experience, professional ranks and titles and had received the training about the ICU-AW (all *P* < 0.05). There were statistical differences in the attitude score of ICU nurses with different age, years of ICU work experience, professional ranks and titles and had received the training about the ICU-AW (all *P* < 0.05). There were statistical differences in the behavior score of ICU nurses with different age, education level, years of ICU work experience, professional ranks and titles and had received the training about the ICU-AW (all *P* < 0.05).

**Table 4:** Univariate analysis on the knowledge, attitude and behavior scores of nurses about ICU-acquired weakness

Items	Knowledge				Attitude				Behavior		
	Score	t/F	P		Score	t/F	P		Score	t/F	P
Gender		7.073	0.006			6.602	0.123			5.169	0.106
Female	22.04 ± 5.21				30.99 ± 51				26.58 ± 5.02		
Male	20.37 ± 5.09				29.94 ± 4.29				26.81 ± 6.72		
Age(y)		5.928	0.013			5.041	0.021			5.267	0.024
18 ∼ 25	20.20 ± 6.01				27.19 ± 6.18				26.04 ± 5.92		
26 ∼ 35	21.41 ± 5.03				30.10 ± 4.98				26.97 ± 6.65		
36 ∼ 45	22.19 ± 5.28				30.49 ± 5.05				27.88 ± 5.35		
> 45	24.43 ± 5.87				31.85 ± 6.12				29.05 ± 5.81		
Education level		6.101	0.029			4.192	0.204			5.1 08	0.004
Junior college degree	19.31 ± 6.44				29.94 ± 4.83				24.07 ± 5.94		
Bachelor degree	21.84 ± 5.16				30.20 ± 5.24				26.94 ± 6.11		
Master degree	23.47 ± 5.07				30.59 ± 5.07				27.89 ± 6.12		
Years of ICU work experience		5.095	0.014			5.608	0.017			5.901	0.023
< 5	19.24 ± 5.89				29.56 ± 5.22				25.26 ± 6.73		
6 ∼ 10	21.02 ± 6.18				30.14 ± 5.87				26.64 ± 5.05		
11 ∼ 20	21.59 ± 5.48				30.97 ± 4.09				27.09 ± 5.94		
> 20	23.09 ± 5.23				31.63 ± 5.25				27.45 ± 5.06		
Professional ranks and titles		7.055	0.031			5.081	0.044			5.036	0.040
Junior nurse	19.62 ± 5.09				29.05 ± 5.31				25.91 ± 6.53		
Senior nurse	20.17 ± 5.95				29.89 ± 4.95				26.49 ± 5.56		
Nurse in charge	21.13 ± 6.67				30.27 ± 5.33				27.01 ± 6.24		
Deputy chief nurse	22.29 ± 5.24				31.85 ± 6.05				27.28 ± 5.85		
Chief nurse	24.85 ± 5.76				31.97 ± 4.77				27.90 ± 5.08		
Marital status		5.844	0.076			5.404	0.112			6.169	0.092
Unmarried	21.05 ± 6.44				30.12 ± 4.69				26.62 ± 5.86		
Married	21.94 ± 5.12				30.42 ± 5.01				53.85 ± 6.02		
Had received the training about the ICU-acquired weakness		5.708	0.001			3.125	0.005			4.099	0.024
Yes	25.03 ± 5.43				31.75 ± 5.48				28.02 ± 6.93		
No	18.95 ± 4.88				29.88 ± 5.73				24.27 ± 6.22		

Notes: t: t test; F, analysis of variance test

### Results of multiple linear regression analysis

As presented in Table 5, multiple linear regression analyses indicated that age, years of ICU work experience, professional ranks and titles, had received the training about the ICU-AW were the influencing factors of knowledge, attitude and behavior of ICU nurses about ICU-AW (all *P* < 0.05).

**Table 5:** Multiple linear regression analysis on the influencing factors of knowledge, attitude and behavior of ICU nurses about ICU-acquired weakness

Variables	Partial regression coefficient	Standard error	Standardized regression coefficient	t	P
Age(y)	5.007	2.413	0.185	2.073	0.021
Years of ICU work experience	6.054	2.302	0.299	2.181	0.004
Professional ranks and titles	4.218	2.166	0.125	3.945	0.016
Had received the training about the ICU-acquired weakness	7.315	2.943	0.344	2.750	0.013

Notes: R^2^ = 0.156, adjusted R^2^ = 0.138, F = 6.597

## Discussion

83.15% of patients with mechanical ventilation > 4 days have ICU-AW, and ICU-AW begin to appear a few hours after mechanical ventilation [23, 24]. Some studies [25–27] have shown that once patients in ICU are complicated with neuromuscular damage, most patients not only cannot be cured, but also their hospitalization and rehabilitation time are significantly prolonged. ICU nurses, as the most direct and frequent contacts of ICU patients, a solid grasp and rational use of ICU-AW knowledge is of great significance to reduce the occurrence of ICU-AW and improve the prognosis of patients [28, 29]. The results of this survey show that nurses in ICU lack relevant knowledge about ICU-AW, nurses’ attitude towards ICU-AW needs to be improved, and nurses’ ICU-AW behavior in ICU needs to be strengthened. Besides, this study has found that age, years of ICU work experience, professional ranks and titles, had received the training about the ICU-AW are the influencing factors of knowledge, attitude and behavior of ICU nurses about ICU-AW, targeted education and interventions are needed to improve the ICU nurses’ ICU-AW knowledge, attitude and behavior.

At present, there are very few studies on ICU-AW in China, which lead to the lack of learning resources and clinical experience of ICU nurses, which is not conducive to the updating of ICU nurses’ knowledge and clinical practice [30]. In general, ICU nurses only rely on their own or other clinical experience to evaluate whether patients have neuromuscular dysfunction and lack systematic knowledge of ICU-AW [31]. The content of critical care textbooks in colleges and universities lags behind and is out of touch with clinical needs, which affects ICU nurses’ understanding of new viewpoints and new knowledge to a certain extent [32]. The results of this study have found that the knowledge score is lower than the attitude and behavior score, it shows that although nurses are lack of knowledge about ICU-AW, their attitudes and behaviors are more positive. It is advisable for nursing managers to continually refresh and expand the theoretical framework pertaining to ICU-AW within the scope of ongoing education and training programs. By increasing the emphasis on ICU-AW specific training modules, a more profound understanding can be instilled within the nursing team. This, in turn, leads to a significant enhancement in the nurses’ knowledge base, attitudes, and behavioral responses towards ICU-AW. Besides, nurses in ICU need to be highly vigilant in their daily nursing work to ensure that all nursing measures are completed timely and correctly, so nurses in ICU are often in a state of tension, which affects their attitude towards ICU-AW to some extent [33]. On the other hand, ICU nurses often work night shifts with heavy load, and high work pressure lead to their physical and mental state of fatigue [34]. Therefore, it is suggested that hospital managers should allocate ICU nursing human resources reasonably, pay attention to the professional psychological state of ICU nurses, and pay attention to the balance between their value and the return, so as to stimulate their work enthusiasm and improve ICU nurses’ attitude towards ICU-AW.

The results of this study have shown that the ICU-AW behavior level of ICU nurses needs to be improved. Some studies [35, 36] have pointed out that ICU generally lacks professional rehabilitation physician, and patients’ early activities generally need to be carried out by ICU nurses, but they lack relevant knowledge of limb rehabilitation exercises, standard procedures for evaluating neuromuscular function and early activity patterns that should be accepted by ICU patients, which can easily lead to poor behavior towards ICU-AW. It is difficult for ICU nurses to carry out early activities for critically ill patients because patients with tracheal intubation or ventilator are unwilling to cooperate because of hard work, while family members are worried about safety [37]. When nursing critically ill patients with changeable and complex conditions, ICU nurses’ relative lack of ICU-AW knowledge directly affects their ICU-AW nursing behavior [38]. Therefore, the ICU-AW behavior of nurses with ICU needs to be strengthened.

There are significant differences in the scores of ICU knowledge among ICU nurses with different ages, working years, educational background and professional title. Previous study [39] has found that ICU nurses with different levels of experience have different degrees of mastery of ICU-AW knowledge, and the higher the educational background, the higher the score of ICU-AW knowledge, which may be that the higher the educational background of ICU nurses, the stronger their curiosity for knowledge, and they are good at observing the premonitory changes of critically ill patients in advance, and analyze and summarize various problems in clinical nursing work to understand and seek solutions. The knowledge score of ICU nurses with senior professional titles is higher than that of primary and intermediate professional titles, which may be related to the strong ability of analyzing and solving problems of these ICU nurses, and they have certain ability of nursing scientific research. When they find that they have doubts about caring for critically ill patients, they may search the relevant literature for solutions. The promotion of professional title is beneficial to the improvement of ICU nurses’ personal professional quality and comprehensive ability [40].

There are some limitations in this survey. First of all, this study is designed for a cross-sectional survey, which only investigate ICU nurses from two tertiary hospitals in China, the sample size of the study is small and limited. Secondly, this survey has only analyzed the general demographic factors that affected the level of ICU-AW knowledge, attitude and behavior of ICU nurses, and does not investigate whether the allocation of ICU nursing human resources, the treatment of ICU nurses and the risk management of critically ill patients have any influence on the results of the study. Besides, there is a lack of official formal training on ICU-AW for the included nurse, the answer regarding had received the training about the ICU-AW may be more subjective. Future large-sample, multicenter and high-quality studies are needed to further analyze and explore the current situation and influencing factors of ICU-AW knowledge, belief and behavior in ICU nures to reduce the clinical ICU-AW.

## Conclusion

In summary, this survey found that the level of knowledge, belief and behavior of ICU nurses towards ICU-AW needs to be improved. Besides, age, years of ICU work experience, professional ranks and titles, had received the training about the ICU-AW are the influencing factors of knowledge, attitude and behavior of ICU nurses to ICU-AW. Hospital nursing managers can carry out targeted intervention and guidance from these aspects to improve the level of ICU-AW knowledge, attitude and behavior of ICU nurses. There is a close relationship among ICU nurses’ ICU-AW knowledge, attitude and behavior. By improving ICU nurses’ awareness of ICU-AW, gradually forming a better belief, and then taking a positive attitude to change ICU-AW behavior, it may be beneficial to reduce the occurrence of ICU-AW and promote the rehabilitation and prognosis of ICU patients.

### Electronic supplementary material

Below is the link to the electronic supplementary material.

Supplementary Material 1

## Data Availability

The data associated with the paper are not publicly available but are available from the corresponding author on reasonable request.

## Abbreviations

ICU-AW: Intensive care unit-acquired weakness

## References

[1] Rahiminezhad E, Zakeri MA, Dehghan M (2023). Muscle strength/intensive care unit acquired weakness in COVID-19 and non-COVID-19 patients. Nurs Crit Care.

[2] Teixeira JP, Mayer KP, Griffin BR, George N, Jenkins N, Pal CA, Gonzalez-Seguel F, Neyra JA (2023). Intensive care unit-acquired weakness in patients with acute kidney Injury: a contemporary review. Am J Kidney Dis.

[3] Diaz Ballve LP, Dargains N, Urrutia Inchaustegui JG, Bratos A, Milagros Percaz M, Bueno Ardariz C, Cagide S, Balestrieri C, Gamarra C, Paz D (2017). Weakness acquired in the intensive care unit. Incidence, risk factors and their association with inspiratory weakness. Observational cohort study. Rev Bras Ter Intensiva.

[4] Zhang W, Tang Y, Liu H, Yuan LP, Wang CC, Chen SF, Huang J, Xiao XY (2021). Risk prediction models for intensive care unit-acquired weakness in intensive care unit patients: a systematic review. PLoS ONE.

[5] Li L, Pan G, Yu L (2023). Research status and prospect of pathogenesis of acquired myasthenia gravis in intensive care unit. Int J Neurol Neurosurg.

[6] Li X, Wu D, Ding X (2022). Research progress on risk prevention, control and prediction model of acquired weakness in intensive care unit. Chin J Mod Nurs.

[7] Garcia-Perez-de-Sevilla G, Sanchez-Pinto Pinto B (2023). Effectiveness of physical exercise and neuromuscular electrical stimulation interventions for preventing and treating intensive care unit-acquired weakness: a systematic review of randomized controlled trials. Intensive Crit Care Nurs.

[8] Klawitter F, Walter U, Axer H, Patejdl R, Ehler J. Neuromuscular Ultrasound in Intensive Care Unit-Acquired weakness: current state and future directions. Med (Kaunas) 2023, 59(5). 10.3390/medicina59050844 PMC10223702 37241077

[9] Yang Z, Wang X, Chang G, Cao Q, Wang F, Peng Z, Fan Y (2023). Development and validation of an intensive care unit acquired weakness prediction model: a cohort study. Front Med (Lausanne).

[10] Huang D, Zhang W, Peng W, Fan Y, He X, Xing R, Yan X, Zhou S, Peng Y, Luo W (2023). Knowledge, attitudes and practices regarding children with ICU-acquired weakness in pediatric intensive care unit among Chinese medical staff: a cross-sectional survey. BMC Nurs.

[11] Castelli L, Iacovelli C, Fusco A, Amoruso V, Cuccagna C, Loreti C, Giovannini S, Padua L. The role of Technological Rehabilitation in patients with intensive care unit weakness: a Randomized Controlled Pilot Study. J Clin Med 2023, 12(7). 10.3390/jcm12072612 PMC10095108 37048695

[12] Cai Y, Chen Y, Sun F. Investigation of ICU acquired weakness in patients with mechanical ventilation and analysis of high risk factors *Modern Medicine* 2020, 16(1): 7–9.

[13] Zhang X, Sun G (2018). Risk factors of acquired weakness in ICU and its influence on short-term and long-term prognosis. PLA J Nurs.

[14] Zhao X, Han J, Ma J (2022). Analysis of muscle strength recovery and influencing factors in patients with acquired weakness in ICU. Nurs Res.

[15] Ye X, Jiang F (2014). Effect of limb functional exercise intensity on rehabilitation effect of patients with ICU. Chin J Nurs.

[16] Wu L. ICU nurses’ knowledge, belief and practice of ICU acquired weakness and analysis of influencing factors Nanchang. Nanchang University; 2016.

[17] Wu L (2017). Investigation on knowledge, belief and behavior of nurses with ICU acquired weakness and analysis of influencing factors.

[18] Xie L, Luo J, Li M, Hu X, Zhu X (2019). Construction of ICU KAP questionnaire and reliability and validity test. J Nurs Adm Manag.

[19] Zheng W, He F (2020). The method of calculating the sample size of current situation survey. Prev Med.

[20] Yuan J (2013). Comparative study on the calculation methods of sample size. Stat decision-making.

[21] Zhu Y, Zheng Y, Yin M (2020). Multiple collinearity test method in statistical analysis. Stat decision-making.

[22] Guo Y, Zhu X, Dai Y (2022). Diagnosis and treatment of multicollinearity in econometrics. China Economic Trade.

[23] Tortuyaux R, Davion JB, Jourdain M (2022). Intensive care unit-acquired weakness: questions the clinician should ask. Rev Neurol (Paris).

[24] Wang W, Xu C, Ma X, Zhang X, Xie P (2020). Intensive care unit-acquired weakness: a review of recent Progress with a look toward the future. Front Med (Lausanne).

[25] Qin ES, Hough CL, Andrews J, Bunnell AE (2022). Intensive care unit-acquired weakness and the COVID-19 pandemic: a clinical review. PM R.

[26] Farhan H, Moreno-Duarte I, Latronico N, Zafonte R, Eikermann M (2016). Acquired muscle weakness in the Surgical Intensive Care Unit: Nosology, Epidemiology, diagnosis, and Prevention. Anesthesiology.

[27] Nunez-Seisdedos MN, Lazaro-Navas I, Lopez-Gonzalez L, Lopez-Aguilera L (2022). Intensive care unit- acquired weakness and hospital functional mobility outcomes following invasive mechanical ventilation in patients with COVID-19: a single-centre prospective cohort study. J Intensive Care Med.

[28] Sun B, Li M (2023). The mechanism, inducement and long-term effects of acquired weakness in intensive care units. Zhonghua Yi Xue Za Zhi.

[29] Akinremi AA, Erinle OA, Hamzat TK (2019). ICU-acquired weakness: a multicentre survey of knowledge among ICU clinicians in South-Western Nigeria. Niger J Clin Pract.

[30] Cai Y, Xu Y, Chen Y (2019). Investigation on knowledge, belief and practice of ICU acquired weakness of nurses in ICU and analysis of influencing factors. J Nurs Adm Manag.

[31] Hou F, Wang J, Li Z (2021). Investigation on knowledge, belief and practice of nurses with acquired weakness of ICU in intensive care unit. Shanghai Nurs.

[32] Feng J, Tian Y, Nie M (2015). Analysis of cognitive status of 253 ICU nurses in a hospital about ICU acquired weakness. J Nurs.

[33] Raurell-Torreda M, Arias-Rivera S, Marti JD, Frade-Mera MJ, Zaragoza-Garcia I, Gallart E, Velasco-Sanz TR, San Jose-Arribas A, Blazquez-Martinez E. group MO: Care and treatments related to intensive care unit-acquired muscle weakness: A cohort study. *Aust Crit Care* 2021, 34(5):435–445. 10.1016/j.aucc.2020.12.005 33663950

[34] Wu Y, Jiang B, Wang G, Wei H, Li B, Shen X, Zhang C, Zhang Z (2020). Current practice and obstacle factors of intensive care unit-acquired weakness assessment. Zhonghua Wei Zhong Bing Ji Jiu Yi Xue.

[35] Wu Y, Zhang Z, Jiang B, Wang G, Wei H, Li B, Shen X, Zhang C (2021). Current practice and barriers to ICU-acquired weakness assessment: a cross-sectional survey. Physiotherapy.

[36] Tang Z, Li F, Zhu Z (2018). Research progress of acquired weakness in ICU. Chin Southwest Natl Med.

[37] Zorowitz RD (2016). ICU-Acquired weakness: a Rehabilitation perspective of diagnosis, treatment, and Functional Management. Chest.

[38] Zhao X, Han J, Ma J (2022). Muscle strength recovery and influencing factors in patients with acquired weakness in ICU. Nurs Res.

[39] Wang Y, Gai Y, Guo X (2023). ICU nurses’ knowledge, belief and practice on acquired weakness of ICU. Gen Pract Nurs.

[40] Van Aerde N, Van den Berghe G, Wilmer A, Gosselink R, Hermans G, Consortium C (2020). Intensive care unit acquired muscle weakness in COVID-19 patients. Intensive Care Med.

