# Supplementary material for: The knowledge, attitude and behavior of ICU nurses regarding ICU-acquired weakness: a cross-sectional survey
Source: BMC Nurs. 2024 Jun 4;23:377. doi: 10.1186/s12912-024-01942-9 (PMC11149312; doi:10.1186/s12912-024-01942-9)
Supplement: Supplementary file 1 — Supplementary Material 1 [file 12912_2024_1942_MOESM1_ESM.docx]

**Questionnaire on knowledge, belief and practice of ICU nurses to ICU acquired weakness**

Dear ICU nurse,

Hello!

Thank you for participating in this survey.

The purpose of this study was to investigate the status quo and main influencing factors of ICU-AW knowledge, attitude and behavior of ICU nurses in Nanjing, to increase nurses' attention to ICU-AW of critically ill patients and improve ICU-AW attitude and behavior of ICU nurses, so as to provide basis for targeted intervention measures to prevent ICU-AW.

This survey is completed anonymously, please fill in truthfully and independently, your answer is for research only, will not affect your current family and work, and we follow the principle of confidentiality.

Note: please mark the number of options you choose with "√". Thank you for your support and contribution to this study!

**Part I**

General information:

1. Age (years); Gender marital status:

2. How many years have you been engaged in nursing work (years).

3. Your working years in ICU (years).

4. Your current education: Junior college degree Bachelor degree Master degree

5. Professional ranks and titles: Junior nurse Senior nurse

Nurse in charge Deputy chief nurse Chief nurse

6. Whether you have been trained for ICU-AW related knowledge: yes no

**Part II**

I. ICU-AW knowledge.

Subjective part:

1. Do you know the concept of ICU-AW？ ①know ②know a little, but not exactly ③don't know

2、Do you know the clinical manifestation of ICU-AW? ①know ②know a little, but not exactly ③don't know

3、Do you know how ICU-AW diagnoses? ①know ②know a little, but not exactly ③don't know

4、Do you know how to evaluate ICU-AW patients? ①know ②know a little, but not exactly ③don't know

5、Do you know what are the risk factors for ICU-AW? ①know ②know a little, but not exactly ③don't know

6、Do you know what preventive measures are available for ICU-AW? ①know ②know a little, but not exactly ③don't know

Objective part:

7、ICU-AW is one of the common complications in critically ill patients.

① Correct ②Uncertain ③ Wrong

8、The incidence of ICU-AW in critically ill patients with mechanical ventilation more than 4 days was 33% and 82%.① Correct ②Uncertain ③ Wrong

9、The diagnosis of ICU-AW mainly depends on the Medical Research Council score (MRC-score). ① Correct ②Uncertain ③ Wrong

10、The main clinical manifestations of ICU-AW patients were difficulty in weaning, paresis or quadriplegia, decreased reflex and muscular atrophy. ① Correct ②Uncertain ③ Wrong

11、ICU-AW will not only prolong the length of stay and increase medical expenses, but also reduce the living ability and survival rate of patients. ① Correct ②Uncertain ③ Wrong

12、Myasthenia gravis and functional impairment are still common in ICU survivors one year after discharge. ① Correct ②Uncertain ③Wrong

13、No movement and exercise may be an important risk factor for ICU-AW. ① Correct ②Uncertain ③Wrong

14、Promoting the activity of ICU patients in the early stage is the most effective intervention to prevent or reduce ICU-AW. ① Correct ②Uncertain ③Wrong

15、Standard insulin therapy can reduce the incidence and duration of neuromuscular complications, thus reducing ICU-AW.。 ① Correct ②Uncertain ③Wrong

II. ICU-AW attitude

1、Do you think your ICU-AW-related knowledge should meet clinical needs? ①Totally agree ②agree ③neutral ④against ⑤Totally against

2、Do you think nurse ICU should dynamically monitor the ICU-AW status of patients? ①Totally agree ②agree ③neutral ④against ⑤Totally against

3、Do you think you should receive formal ICU-AW nursing training? ①Totally agree ②agree ③neutral ④against ⑤Totally against

4、Do you think nurses should be responsible for evaluating ICU-AW nursing?

①Totally agree ②agree ③neutral ④against ⑤Totally against

5、Do you think early functional exercise is very important for the prevention and recovery of ICU-AW? ①Totally agree ②agree ③neutral ④against ⑤Totally against

6、Do you think health care workers should pay attention to the prevention of ICU-AW as much as other symptoms (such as delirium)? ①Totally agree ②agree ③neutral ④against ⑤Totally against

7、Do you think it is necessary to educate patients or their families about ICU-AW in clinical work? ①Totally agree ②agree ③neutral ④against ⑤Totally against

8、Do you think the ICU-AW status of critically ill patients should be included in the shift in clinical work? ①Totally agree ②agree ③neutral ④against ⑤Totally against

III. ICU-AW behavior

1、Do you actively pay attention to the ICU-AW status of patients in your clinical work? ①always ②yes ③occasionally④very few ⑤never

2、Do you communicate with patients about limb muscle strength in clinical work? ①always ②yes ③occasionally④very few ⑤never

3、Do you evaluate the ICU-AW of patients in clinical work? ①always ②yes ③occasionally④very few ⑤never

4、Will you report the patient's muscle strength to the doctor in your department in time? ①always ②yes ③occasionally④very few ⑤never

5、Will you provide effective early functional exercise for critically ill patients? ①always ②yes ③occasionally④very few ⑤never

6、Do you instruct your family members to help patients with appropriate activities to relieve symptoms such as physical fatigue? ①always ②yes ③occasionally④very few ⑤never

7、Do you evaluate the nursing intervention of patients' early activities in time? ①always ②yes ③occasionally④very few ⑤never

8、Do you accumulate ICU-AW related knowledge in the course of your work? ①always ②yes ③occasionally④very few ⑤never
